# Use of Laser Assisted Optical Rotational Cell Analyzer (LoRRca MaxSis) in the Diagnosis of RBC Membrane Disorders, Enzyme Defects, and Congenital Dyserythropoietic Anemias: A Monocentric Study on 202 Patients

**DOI:** 10.3389/fphys.2018.00451

**Published:** 2018-04-27

**Authors:** Anna Zaninoni, Elisa Fermo, Cristina Vercellati, Dario Consonni, Anna P. Marcello, Alberto Zanella, Agostino Cortelezzi, Wilma Barcellini, Paola Bianchi

**Affiliations:** ^1^UOC Oncoematologia, UOS Fisiopatologia delle Anemie, Fondazione IRCCS Ca' Granda Ospedale Maggiore Policlinico, Milan, Italy; ^2^UO Epidemiologia, Fondazione IRCCS Ca' Granda Ospedale Maggiore Policlinico, Milan, Italy; ^3^Università degli Studi di Milano, Milan, Italy

**Keywords:** red cell disorders, chronic hemolytic anemias, ektacytometer, LoRRca MaxSis, red cell membrane defects, red cell metabolism, differential diagnosis

## Abstract

Chronic hemolytic anemias are a group of heterogeneous diseases mainly due to abnormalities of red cell (RBC) membrane and metabolism. The more common RBC membrane disorders, classified on the basis of blood smear morphology, are hereditary spherocytosis (HS), elliptocytosis, and hereditary stomatocytoses (HSt). Among RBC enzymopathies, the most frequent is pyruvate kinase (PK) deficiency, followed by glucose-6-phosphate isomerase, pyrimidine 5′ nucleotidase P5′N, and other rare enzymes defects. Because of the rarity and heterogeneity of these diseases, diagnosis may be often challenging despite the availability of a variety of laboratory tests. The ektacytometer laser-assisted optical rotational cell analyser (LoRRca MaxSis), able to assess the RBC deformability in osmotic gradient conditions (Osmoscan analysis), is a useful diagnostic tool for RBC membrane disorders and in particular for the identification of hereditary stomatocytosis. Few data are so far available in other hemolytic anemias. We evaluated the diagnostic power of LoRRca MaxSis in a large series of 140 patients affected by RBC membrane disorders, 37 by enzymopathies, and 16 by congenital diserythropoietic anemia type II. Moreover, nine patients with paroxysmal nocturnal hemoglobinuria (PNH) were also investigated. All the hereditary spherocytoses, regardless the biochemical defect, showed altered Osmoscan curves, with a decreased Elongation Index (EI) max and right shifted Omin; hereditary elliptocytosis (HE) displayed a trapezoidal curve and decreased EImax. Dehydrated hereditary stomatocytosis (DHSt) caused by *PIEZO1* mutations was characterized by left-shifted curve, whereas *KCNN4* mutations were associated with a normal curve. Congenital diserythropoietic anemia type II and RBC enzymopathies had Osmoscan curve within the normal range except for glucosephosphate isomerase (GPI) deficient cases who displayed an enlarged curve associated with significantly increased Ohyper, offering a new diagnostic tool for this rare enzyme defect. The Osmoscan analysis performed by LoRRca MaxSis represents a useful and feasible first step screening test for specialized centers involved in the diagnosis of hemolytic anemias. However, the results should be interpreted by caution because different factors (i.e., splenectomy or coexistent diseases) may interfere with the analysis; additional tests or molecular investigations are therefore needed to confirm the diagnosis.

## Introduction

Hereditary red cell membrane disorders and defects of RBC metabolism are a group of rare and heterogeneous disorders eases characterized by chronic hemolytic anemia of variable degree, jaundice, and splenomegaly. The diagnostic workflow is based on laboratory tests, clinical examination, personal, and family history, and on the exclusion of other causes of hemolysis. However, because of their rarity and wide clinical heterogeneity, the diagnosis of these defects is often difficult, particularly in mild and atypical forms.

RBC membrane disorders are caused by defects of cytoskeleton proteins, which form a complex network providing the erythrocyte with its shape and deformability. Consequently, abnormalities of single proteins may impair the structural and functional integrity of the entire membrane causing an alteration of RBC shape. In general, defects in spectrin, ankyrin, protein band 3, and band 4.2 weaken the cohesion between the membrane lipid bilayer and cytoskeleton, leading to the release of microvesicles, loss of surface, and transformation of the discocyte into a spherocyte: these alterations result in hereditary spherocytosis (HS). Conversely, hereditary elliptocytosis (HE) is due to impaired interaction of spectrin dimers or defective spectrin-actin-protein 4.1 complex. If cytoskeleton weakening is excessive, red blood cells can undergo severe deformations resulting in hereditary pyropoikilocytosis (HPP), which mimicks cell fragmentation due to heat exposure (Mohandas and Gallagher, 2008; Perrotta et al., 2008).

The hereditary stomatocytoses (HSt) are caused by defects of membrane cation permeability and cell volume regulation; they are divided in two different entities: dehydrated hereditary stomatocytosis (DHSt) due to mutations in *PIEZO1* and *KCNN4* genes, and overhydrated hereditary stomatocytosis (OHS) associated with mutations in *RhAG* gene; additional more rare forms of stomatocytosis include defects in *ABCB6* (pseudohyperkaliemia) or *GLUT1* (cryohydrocytosis with neurological impairment) (Glogowska and Gallagher, 2015; Badens and Guizouarn, 2016).

The laboratory diagnosis of HS and other RBC membrane disorders is usually based on indirect assays that investigate the surface area-to-volume ratio, typically reduced in spherocytes, such as NaCl osmotic fragility tests (on fresh and incubated blood), Glycerol Lysis (GLT) and Acidified Glycerol Lysis tests (AGLT), and Pink test. These methods do not differentiate HS from secondary spherocytosis and may result falsely negative in some HS cases, particularly the mildest ones. The direct flow cytometric EMA-binding test (King et al., 2000), shows high sensitivity and specificity (Bianchi et al., 2012). In severe or atypical HS, the diagnostic workflow may require SDS-PAGE quantification of RBC membrane proteins (King et al., 2015) or more recently molecular characterization of the defect through NGS platforms (He et al., 2017). SDS-PAGE analysis is also necessary in differential diagnosis of congenital dyserythropoietic anemia type II (CDAII) (King and Zanella, 2013).

Chronic hereditary hemolytic anemias are also due to enzymatic defects of erythrocyte metabolism. Metabolic energy is needed to maintain the red cell shape, to keep the iron of hemoglobin in the divalent form, to pump ions against electrochemical gradients and to maintain the sulfydryl groups of proteins in the active, reduced forms. Pyruvate kinase (PK) deficiency is the most common ezymopathy associated with chronic hemolytic anemia, followed by glucosephosphate isomerase (GPI) and pyrimidine 5′-nucleotidase (P5′N) deficiencies (Koralkova et al., 2014). The diagnosis of these disorders relies on the exclusion of the more common hemolytic anemias, ultimately depending on the determination of red cell enzyme activity by quantitative assays and identification of the molecular defect in the causative genes. However, except for the rare enzymopathies whose causative genes have an ubiquitous expression leading to non-hematological signs (myopathy and neuromuscular abnormalities in triosephosphate isomerase (TPI) or phosphoglycerate kinase (PGK) deficiency), the clinical picture of these diseases is similar to the other congenital hemolytic anemias, making in some cases differential diagnosis tricking with possible misdiagnosis.

Osmotic gradient ektacytometry, which measures red cell deformability, osmotic fragility and cell hydration, has long been considered a reference technique for diagnosis of RBC membrane disorders, particularly HSt. Ektacytometry was however rarely used as a routine diagnostic tool due to the limited availability of the original device. Recently, a new generation ektacytometer has been made available, the Laser-assisted Optical Rotational Cell Analyzer (LoRRca), which is proposed as a simple laboratory method to detect red cell membrane abnormalities (Da Costa et al., 2013, 2016; Lazarova et al., 2017; Llaudet-Planas et al., 2018). Few data are so far available in other hemolytic anemias.

In this study we evaluated the performance and the diagnostic power of the LoRRca MaxSis in a large series of 202 consecutive patients with different forms of chronic hemolytic anemias referred to our Institution over the period 2014–2017, with the aim of ascertaining the possible role of this tool in the workflow of a laboratory specialized in the diagnosis of these diseases.

## Materials and methods

### Patients

A total of 202 consecutive patients with chronic hemolytic anemias (102 males and 100 females, median age 29 years, range 0.3–82 years) were diagnosed from 2014 to 2017. Peripheral blood was collected from patients and controls during diagnostic procedures after obtaining informed consent and approval from the Institutional Human Research Committee. The procedures followed were in accordance with the Helsinki international ethical standards on human experimentation. The great majority of samples were collected in our Institute; samples from other centers were shipped maintaining a temperature of 4°C and processed within 24 h. All tests were performed in a single site. None of the patients had been transfused within the 3 months preceding the study.

### Testing for diagnosis of chronic hemolytic anemias

The diagnostic workflow for chronic hemolytic anemias includes RBC morphology, complete blood count, hemolysis markers, EMA binding test, osmotic fragility tests, and RBC enzymes activities. Confirmatory tests include SDS-PAGE analysis of red cell membrane proteins, and molecular testing for hereditary stomatocytosis, CDAII and enzymopathies.

**Figure 1:**
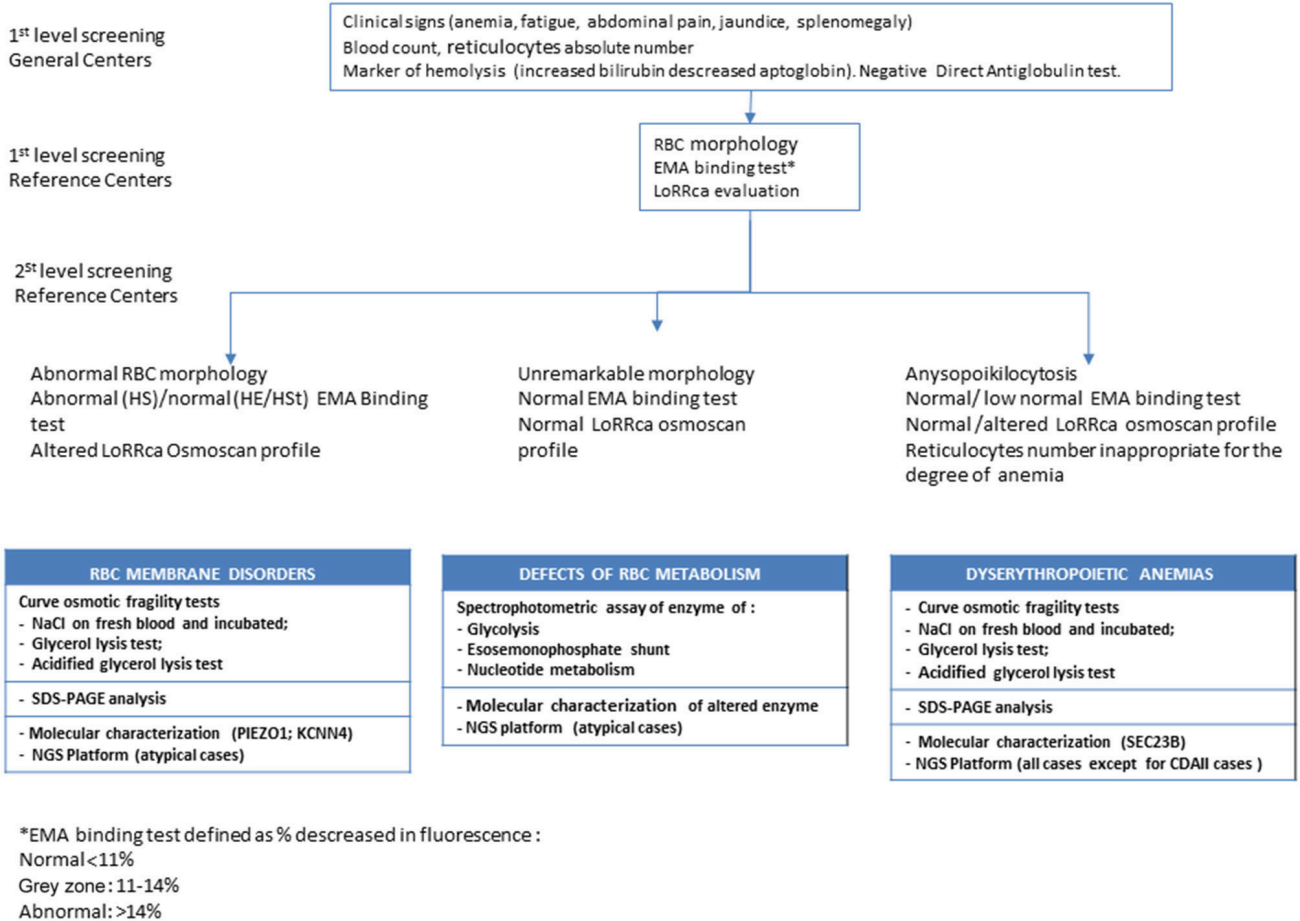
Diagnostic workflow for chronic hemolytic anemias.

Routine hematological investigations were carried out according to Dacie and Lewis (2001). The RBC morphology was assessed by two independent and expert operators. Red cell osmotic fragility was evaluated by the following tests: NaCl osmotic fragility test on both fresh and incubated blood, standard GLT, AGLT, and Pink test. EMA-binding test was performed as described by King et al. (2000) with minor modifications (Bianchi et al., 2012). Since 2014 Osmoscan LoRRca analysis was included in the diagnostic panel and performed in all the patients referred to our Institute to confirm the diagnosis of hemolytic anemia. The diagnostic workflow adopted for chronic hemolytic anemias is reported in Figure 1.

Red cell membrane proteins were analyzed by SDS-PAGE according to Fairbanks et al. (1971) and Laemmli (1970); on the basis of SDS-PAGE analysis HS patients were stratified according to their membrane defects on the following groups: deficiency of band 3, spectrin, ankyrin or combined spectrin/ankyrin, and no detectable defect (Mariani et al., 2008).

RBC enzymes activities were determined according to Beutler (1984); diagnosis of a red cell enzyme defect was confirmed at molecular level by DNA sequencing (*PK-LR* gene for PK deficiency, *GPI* gene for GPI deficiency, *NT5C3A* for P5′N, *TPI1* for TPI). Molecular testing was performed also in CDAII (*SEC23B* gene) and hereditary stomatocytosis patients (*PIEZO1* and *KCNN4* genes).

### Osmotic gradient ektacytometry (osmoscan curve)

Two hundred and fifty microliters of EDTA sample suspended in 5 mL of polyvinylpyrrolidone buffer (PVP, Mechatronics, Hoorn, The Netherlands) were used for the analysis. Osmoscan was performed by means of Laser-assisted Optical Rotation Cell Analyzer (LoRRca MaxSis, Mechatronics, Hoorn, The Netherlands) according to the manufacturer's instructions and as reported in detail by Da Costa et al. (2013, 2016). The osmotic gradient curves reflect RBC deformability as a continuous function of suspending medium osmolality. The following parameters were evaluated: the Omin-value corresponds to the osmolality at which the deformability reaches its minimum and represents the 50% of the RBCs hemolysis in conventional osmotic fragility assays, reflecting mean cellular surface-to-volume ratio; the elongation index (EI) max corresponds to the maximal deformability or elongation obtained near the isotonic osmolality and is an expression of the membrane surface; the Ohyper (the osmolality in the hypertonic region corresponding to 50% of the EImax) reflects mean cellular hydration status; the area under the curve (AUC), is defined in the provided software as the AUC beginning from a starting point in the hypo-osmolar region and an ending point in the hyper-osmolar region (instrument settings 500 mOsm/kg; Baskurt and Meiselman, 2004; Baskurt et al., 2009). The corresponding parameters on the X or Y axis of Omin (EImin), EImax (Omax), and Ohyper (EIhyper), respectively, were also analyzed.

### Osmoscan analysis in normal subject

We evaluated Osmoscan curve of 170 healthy blood donors. All the main parameters of the curve showed a Gaussian distribution, therefore the area covered by all the control curves was considered as the reference range. Osmoscan profile obtained from each patient was compared with the reference range curve and with a daily normal control analyzed together with the sample.

### Statistical analysis

We calculated Sperman's rho correlation coefficients of osmoscan parameters and hematologic data either considering the total number of patients (*n* = 202), independently from the disease, either in HS/not HS patients. Osmoscan parameters across diseases were compared with Kruskal-Wallis tests. Receiver operating characteristic (ROC) analysis was used to calculate the Omin cut-off to discriminate HS between normal control and patients with other membrane defects. We also calculated sensitivity (Se) and specificity (Sp) at optimal cut-offs calculated according to the non-parametric approach proposed by Liu (2012), implemented in the Stata command “cutpt.” Statistical analyses were performed with Stata 15 (StataCorp, 2017).

## Results

### Patients

On the basis of these criteria 116 patients were diagnosed as HS and 86 as hemolytic anemias other than HS: 15 HE, 9 HSt (6 *PIEZO1* and 3 *KCNN4* variants), 37 erythroenzymopaties (27 PK, 4 P5′N, 1 TPI, and 5 GPI deficiency), 16 CDAII. Nine patients underwent further investigations and were diagnosed as paroxysmal nocturnal hemoglobinuria (PNH). At the time of the study, 39 patients were splenectomized and 154 non-splenectomized. Hematologic and laboratory data of the patients grouped on the basis of their pathology and presence or not of spleen are reported in Table 1.

**Table 1:** Hematological characteristics of 202 consecutive patients with chronic hemolytic anemias divided according to disease.

**SPLENECTOMY**		**Hb (gr/dL)**	**Reticulocyte (x10^9^/L)**	**MCV (fL)**	**MCH (pg/cell)**	**MCHC (gr/dL)**	**RDW (%)**	**Uncojugated bilirubin (mg/dL)**	RBC morphology^**^ (%)	**EMA (% reduction)**
		**(F 12–16; M 13.5–17.5)**	**(24–84)**	**(78–99)**	**(25–35)**	**(31–37)**	**(11.8–14.8)**	**(<0.8)**	**Na**	**(<11%)**
HS	NO	12	266	84	29.7	35.9	18.4	1.95	9	−29
	(*N* = 92)	(6.3/16.4)	(35/649)	(69/95.4)	(23.5/34.7)	(30.1/40.1)	(11.4/26.3)	(0.5/10.1)	(2/59)	(−62/+2)
	YES	15.7	145	86.6	32.3	35.7	13	0.71	7.5	−29
	(*N* = 16)	(9.3/17.6)	(51/362)	(75/97.3)	(30.4/33.1)	(29.4/38)	(11.1/19.7)	(0.2/9.3)	(1/19)	(−38/−16)
HE	NO	13.8	59	84	29.5	35	13.4	0.9	43	−8
	(*N* = 15)	(9.8/15.9)	(31/260)	(56.3^*^/92.2)	(19.3/32)	(33.1/36.4)	(11.8/17.5)	(0.4/1.4)	(10/90)	(−14/+4)
	YES	Na	Na	Na	Na	Na	Na	Na	Na	Na
	(*N* = 0)									
HSt	NO	9.9	260	102	36.7	35.7	16	3.2	8	+6
	(*N* = 5)	(8.3/13)	(104–273)	(71.9^*^/110)	(24.8/39.2)	(34.5/37.8)	(13.9/18.6)	(1.7/4.8)	(6/22)	(−5/+15)
	YES	11.7	272.5	105	37.5	35	16	1.7	13	+22
	(*N* = 4)	(11/13.2)	(166/654)	(101/109)	(34.1/40.1)	(33.5/38.3)	(15.1/19.7)	(0.8/6.7)	(6/33)	(+5/+30)
PK, P5′N,	NO	10.3	171	94	32.8	34.3	13.6	2.2	Na	+7
	(*N* = 26)	(5/14.5)	(53/727)	(67.6^*^/108)	(23.1/36.7)	(28.8/36.3)	(10.9/21.4)	(0.5/9.6)		(−9/+22)
TPI, GPI	YES	9.3	508	120	37.8	32.6	15.3	5.2	Na	+23
	(*N* = 11)	(7.5/12)	(88/1740)	(85.3/127)	(27.8/41.1)	(31.6/33.5)	(12.7/24.7)	(1.3/13.5)		(+19/+33)
CDAII	NO	10.7	92.5	86.4	30	35	19.4	2.2	Na	−13
	(*N* = 9)	(7.4/12.7)	(64/121)	(75.2/94)	(25.3/31.6)	(32.9/38.2)	(13.4/24)	(1/3.8)		(−19/+17)
	YES	10.1	40	94	33.6	33.7	20	1.7	Na	−20.5
	(*N* = 7)	(8.3/11.3)	(35/63)	(83.4/96.7)	(27.4/33.7)	(31.2/34.9)	(17.1/29.7)	(0.7/5.7)		(−28/−11)
PNH	NO	10.6	97	93.7	32	32.6	15.7	0.9	Na	+3
	(*N* = 9)	(7.9/13.6)	(52/248)	(81.3/111)	(27.7/34.4)	(31.1/34.9)	(12.8/20)	(0.5/1.4)		(+1/+16)
	YES	Na	Na	Na	Na	Na	Na	Na	Na	Na
	(*N* = 0)									

*Values are expressed as median (range); Hb, hemoglobin, MCV, mean cell volume; MCH, mean corpuscular hemoglobin; MCHC, mean corpuscular hemoglobin concentration; RDW, red cell distribution width; RBC, red blood cell; EMA, EMAbinding test; Na, not available*.

* *beta-trait*.

** *% spherocytes for HS; % elliptocytes for HE; % stomatocytes for HSt*.

### Osmoscan curve in chronic hemolytic anemias

**Figure 2:**
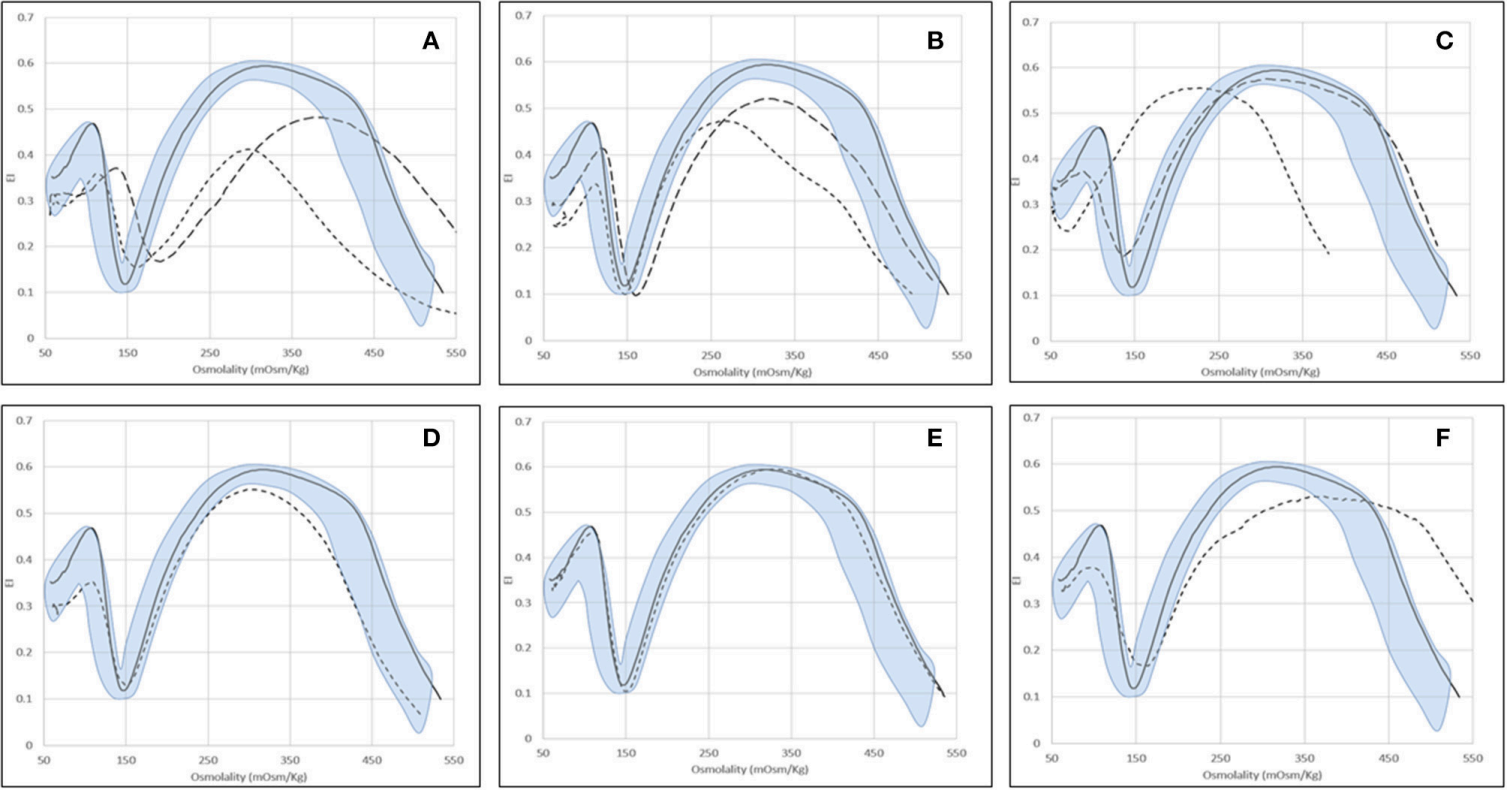
Examples of typical osmoscan profiles in hemolytic anemias. Continuous line represents a daily control and shaded area the control range curve. **(A)** HS = hereditary spherocytosis: HS1 (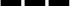), HS2 (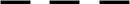); **(B)** HE = hereditary elliptocytosis: HE1 (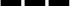), HE2 (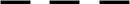); **(C)** HSt = hereditary stomatocytosis: HSt-PIEZO1 (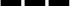), HSt-KCNN4 (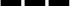); **(D)** CDAII (non-splenectomised) = congenital diserythropoietic anemia type II; **(E)** PKD (non-splenectomised) = pyruvate kinase deficiency; **(F)** GPI = glucosephosphate isomerase deficiency.

Typical Osmoscan profiles obtained in different kind of hemolytic anemias are reported in Figure 2; the median values and range of specific parameters are summarized in Table 2 for all the patients and normal controls. To reach a significant volume of data for statistical analysis some enzymopathies (PK, P5′N, and TPI deficiency) were grouped together since they didn't show significant differences of Osmoscan parameters (data not shown). Patients affected by GPI deficiency were separately considered due to typical abnormalities of Osmoscan curve.

**Table 2:** Osmoscan parameters in normal controls and in patients with chronic hemolytic anemias (splenectomized patients with enzymopathies and CDAII were excluded)^§^.

	***N***	**Omin**	**EImax**	**Ohyper**	**EImin**	**Omax**	**EIhyper**	**AUC**
		**(mOsm/kg)**		**(mOsm/kg)**		**(mOsm/kg)**		
HS	Total	166	0.53	444	0.12	334	0.26	108
	(*N* = 116)	(144–201)^**^	(0.41–0.59)^**^	(389–547)^**^	(0.08–0.19)^*^	(285–409)^**^	(0.21–0.29)^**^	(82–131)^**^
HE	Total	154	0.53	471	0.1	312	0.27	119
	(*N* = 15)	(130–178)^**^	(0.47–0.59)^**^	(428–526)	(0.08–0.19)^*^	(268–339)	(0.24–0.3)^**^	(106–157)^**^
HSt (PIEZO1)	Total	98	0.58	394	0.18	265	0.29	146
	(*N* = 6)	(71–136)^**^	(0.53–0.61)	(342–416)^**^	(0.14–0.24)^**^	(211–299)^**^	(0.27–0.3)	(130–153)
HSt (KCNN4)	Total	157	0.52	510	0.2	338	0.28	133
	(*N* = 3)	(146–161)	(0.51–0.58)^*^	(483–517)	(0.17–0.22)^*^	(303–341)	(0.26–0.29)	(126–144)
PK, P5′N, TPI	Not splenect	144	0.6	482	0.13	313	0.3	149
	(*N* = 24)	(122–173)	(0.56–0.61)	(427–565)^**^	(0.1–0.23)^**^	(253–368)	(0.28–0.31)	(138–161)
GPI	Total	157	0.56	552	0.16	372	0.28	132
	(*N* = 5)	(146–176)^**^	(0.51–0.58)^*^	(500–579)^**^	(0.11–0.2)^*^	(315–381)^*^	(0.26–0.3)^*^	(117–146)^*^
CDAII	Not splenect	152	0.58	458	0.13	327	0.29	133
	(*N* = 9)	(133–164)^*^	(0.55–0.59)^**^	(432–492)	(0.11–0.16)	(318–329)^**^	(0.28–0.3)^**^	(119–145)^**^
PNH	Total	150	0.6	492	0.13	323	0.3	146
	(*N* = 9)	(132–164)	(0.58–0.6)	(457–538)^*^	(0.11–0.15)^*^	(301–357)^*^	(0.29–0.3)	(132–156)
Controls	Total	142	0.6	464	0.12	311	0.3	148
	(*N* = 170)	(125–160)	(0.46–0.62)	(418–512)	(0.09–0.3)	(273–355)	(0.29–0.31)	(114–168)
	MEAN ± 1 SD	142 ± 6.8	0.6 ± 0.01	464.5 ± 15.45	0.12 ± 0.02	310.6 ± 12.15	0.3 ± 0.02	147.8 ± 6.6

*Values are expressed as median (range)*.

* p < 0.05;

** *p < 0.001 vs. controls*.

§ *Only non-splenectomized patients affected by enzymopathies (PK and P5′N) and CDAII were considered due to differences in Osmoscan curve shape observed in the splenectomized ones*.

Osmoscan curves were analyzed in splenectomized and non-splenectomized patients; significant differences in shape were observed particularly in enzyme deficiencies and CDAII patients (see below), therefore for these diseases only data from non-splenectomized patients are reported in Table 2.

The results of Osmoscan parameters were correlated with hematologic data, in particular RBC morphology (number of spherocytes, elliptocytes, or stomatocytes), red cell indexes (MVC, MCHC), osmotic fragility tests (GLT, AGLT), and EMA binding test (Table 3 and Supplementary Figure 1). Omin showed a negative correlation with GLT, AGLT, and a positive one with EMA-binding test. Moreover, Omin was associated with results of incubated osmotic fragility test (NaCl inc) being 137 ± 28.7, 154 ± 11.5, and 166 ± 12.6 mOsm/kg in patients with decreased, normal or increased osmotic fragility, respectively (*p* < 0.0001). Interestingly, EImax was positively correlated with EMA binding test results; Ohyper showed a negative correlation with MCHC. Finally, AUC was correlated with RBC parameters and osmotic fragility tests, as expected being the resultant of Osmoscan parameters.

**Table 3:** Correlation analysis among Osmoscan parameters, Osmotic fragility tests, EMA-binding and red blood cell indexes in patients with chronic hemolytic anemias.

		**RDW**	**MCV**	**MCHC**	**Retic**	**AGLT**	**GLT**	**EMA**
Omin	*r*	0.033	−0.236	0.308	0.266	−**0.658**	−**0.545**	−**0.589**
	*P*	0.72	0.008	0.0005	0.003	<0.0001	<0.0001	<0.0001
Eimax	*r*	−0.210	0.247	−0.167	−0.091	0.316	0.298	**0.419**
	*P*	0.02	0.006	0.06	0.31	0.0004	0.0008	<0.0001
Ohyper	*r*	0.075	0.160	−**0.585**	0.014	0.052	0.052	0.205
	*P*	0.40	0.08	<0.0001	0.875	0.57	0.57	0.022
AUC	*r*	−0.095	**0.416**	−**0.506**	−0.262	**0.554**	**0.542**	**0.648**
	*P*	0.30	<0.0001	<0.0001	0.003	<0.0001	<0.0001	<0.0001

*Spearman' rho correlation analysis was performed in 124 patients for whom all the considered parameters were available*.

*r, correlation coefficient; P, p-values; GLT, standard glycerol lysis test; AGLT, acidified glycerol lysis test; EMA, eosin-5-maleimide. Highly significant correlations are reported in bold*.

#### Hereditary spherocytosis

The typical Osmoscan curve in HS patients is reported to be characterized by a decreased EImax, a shift to the right of Omin and to the left of Ohyper and, consequently, a decreased AUC. As shown in Table 2, differences in all these parameters were observed when comparing HS patients with normal controls (*p* < 0.001). All the patients showed decreased AUC (referring to mean ± 2 SD of controls), all but two decreased EImax, the majority (91%) had increased Omin, but only 31% had decreased Ohyper; 27 patients showed abnormalities in all the four parameters. Interestingly, 12/116 HS patients showed increased Ohyper generating a second kind of osmoscan profile for HS (Figure 2A). No differences were observed when patients were stratified according to splenectomy, to the type of biochemical defect (spectrin, spectrin/ankyrin, band 3, 4.2 protein deficiency), or to the severity of anemia.

#### Hereditary elliptocytosis

Due to the elongated shape and decreased deformability, the resulting Osmoscan analysis in HE is characterized by a trapezoidal curve that is considered a typical finding in this disease. In the analyzed series (Table 2), HE patients (*n* = 15) displayed increased Omin (median 154, range 130–178), decreased EImax (median 0.53, range 0.47–0.59), and AUC (median 119, range 106–157) (*p* < 0.001 for all). Typical trapezoidal shape was observed only in nine out of the 15 analyzed patients; in the remaining six cases (including the three patients with protein 4.1 defect) the curve fell in the area covered by HS patients (Figure 2B) making not possible a differential diagnosis among these two diseases by Osmoscan only. The shape of the curve was independent from the amount of ovalo/elliptocytes, ranging from 10 to 90%.

#### Hereditary stomatocytosis

Six out of the nine HSt patients analyzed had mutations in *PIEZO1* gene and showed an Osmoscan curve characterized by normal AUC and EImax, increased EImin (median 0.18, range 0.14–0.24) and significant left-shift of both Omin (median 98, range 71–136) and Ohyper (median 394, range 342–416, *p* < 0.001, for both) (Figure 2C). On the contrary, the remaining three patients, presenting R352H mutation in *KCNN4* gene, displayed Osmoscan parameters comparable to normal controls (Table 2, Figure 2C).

#### Congenital dyserythropoietic anemia type II

The Osmoscan curve of the nine non-splenectomized cases showed increased Omin (median 152, range 133–164, *p* < 0.05), decreased EImax (0.58, range 0.55–0.59, *p* < 0.001), and AUC (median 133, range 119–146, *p* < 0.001); Ohyper-values were comparable with controls (Table 2). Despite of this, the Osmoscan curve falls for some parameters inside the normal control area (three patients had normal Omin and four normal EImax), not allowing to identify a diagnostic shape in CDAII (Figure 2D). Moreover, differences in Osmoscan parameters were observed in splenectomized patient (see below).

#### Enzyme defects

The 24 non-splenectomized patients affected by PK (20 cases), Pyr5′N (3 cases), and TPI deficiency (1 case) displayed normal Osmoscan curve, with the only exception of increased Ohyper-values in some cases (median 482, range 427–565, *p* < 0.001; Table 2). Statistical differences were maintained in the PK deficient patients when separately analyzed. It is worth noting that all the five GPI deficient cases showed a striking enlargement of the right segment of the curve, which appears to be interrupted at 500 mOsm/Kg (Figure 2F). Although the number of patients is limited, a significantly increased Omin (median 157, range 146–176, *p* < 0.001) and, even more, Ohyper (median 552, range 500–579, *p* < 0.001) was observed; on the contrary, EImax was decreased compared to controls (median 0.56, range 0.51–0.58, *p* < 0.05) (Table 2).

#### Paroxysmal nocturnal hemoglobinuria

No differences were observed with normal controls except for increased OHyper-values (median 492, range 457–538, *p* < 0.05; Table 2).

#### Effect of splenectomy and concomitant defects on osmoscan curve

**Figure 3:**
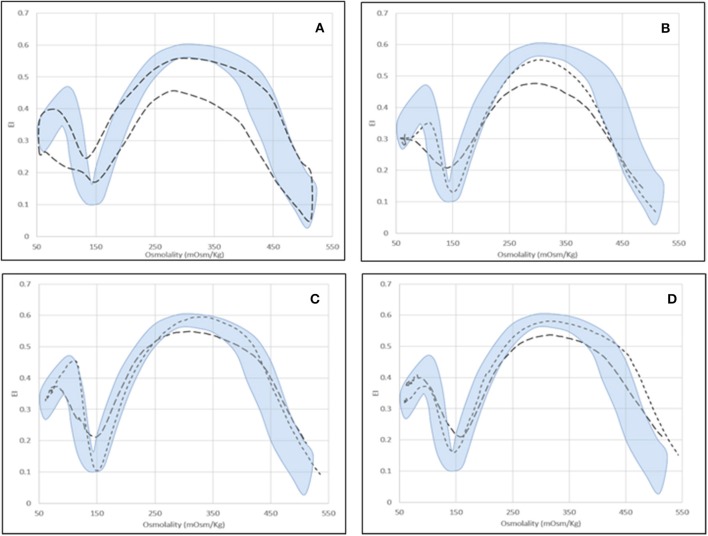
Osmoscan profile in splenectomized patients. **(A)** Shaded area = control range curve; Dashed area = splenectomised patients (seven affected by PKD, two by Pyr5′N deficiency and seven by CDAII); **(B)** CDAII: non-splenectomised (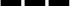), splenectomised (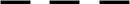); **(C)** PKD: non-splenectomised (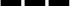), splenectomised (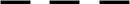); **(D)** Pyr5′N deficiency: non-splenectomised (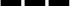), splenectomised (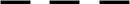).

For some diseases (i.e., enzyme defects and CDAII), Osmoscan curves of splenectomized patients fall into a defined area irrespective of the pathology, characterized by significantly decreased EImax and AUC and increased EImin and EIhyper (*p* < 0.05; Table 4, Figures 3A–D).

**Table 4:** Osmoscan parameters in splenectomized and non-splenectomized patients.

	**Splenectomy**	**Omin**	**EImax**	**Ohyper**	**EImin**	**Omax**	**EIhyper**	**AUC**
		**(mOsm/kg)**		**(mOsm/kg)**		**(mOsm/kg)**		
Enzyme defect	Total	146	0.59	485	0.13	311	0.3	147
		(122–173)	(0.54–0.61)	(427–565)	(0.1–0.23)	(253–368)	(0.27–0.31)	(132–161)
	NO	144	0.6	482	0.13	313	0.3	149
	(*N* = 24)	(122–173)	(0.56–0.61)^*^	(427–565)	(0.1–0.23)^*^	(253–368)	(0.28–0.31)^*^	(138–161)^*^
	YES	147	0.56	490	0.17	309	0.28	144
	(*N* = 8)	(132–158)	(0.54–0.6)	(472–501)	(0.12–0.21)	(286–325)	(0.27–0.3)	(132–152)
CDAII	Total	148	0.55	456	0.16	319	0.28	130
		(119–164)	(0.47–0.59)	(432–492)	(0.11–0.25)	(265–356)	(0.23–0.3)	(113–145)
	NO	152	0.58	458	0.13	327	0.29	133
	(*N* = 9)	(133–164)	(0.55–0.59)^*^	(432–492)	(0.11–0.16)^*^	(318–329)^*^	(0.28–0.30)^*^	(119–145)^*^
	YES	140	0.49	449	0.21	287	0.25	121
	(*N* = 7)	(119–156)	(0.47–0.51)	(438–466)	(0.18–0.25)	(235–306)	(0.23–0.26)	(113–131)

*Values are expressed as median (range). Enzyme defect included = PK, P5′N, and TPI deficiency*.

* *p < 0.05 splenectomised vs. non-splenectomised*.

Moreover, concomitant defects may also interfere with Osmoscan analysis. In the analyzed series all the three cases carrying an additional β-thalassemia trait showed a left-shifted Osmoscan curve, representing a possible cause of misdiagnosis with dehydrated stomatocytosis.

### Scatter plot analysis of osmoscan parameters

**Figure 4:**
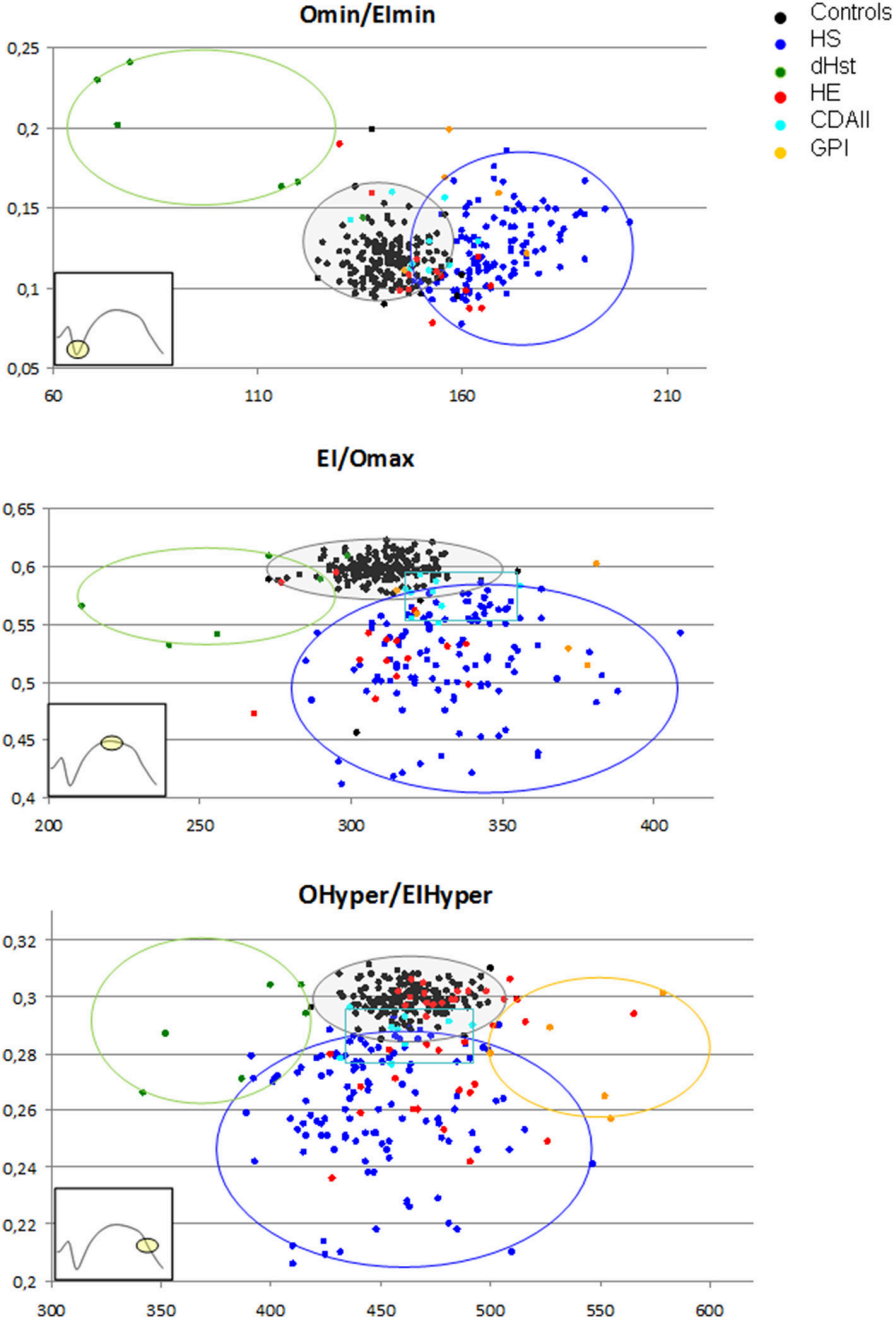
Osmoscan coupled parameters differently clustered depending on diseases. Controls (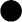), HS (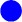), CDA II (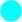), HE (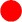), HSt (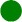), GPI (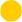).

As reported in Table 2, overlaps are present among data obtained from HS, CDAII, and HE patients, doesn't allowing differential diagnosis by the analysis of the main Osmoscan parameters alone. A scatter plot analysis of Omin, EImax, and Ohyper coupled with the corresponding x-, y-axis-values (EImin, Omax, and EIhyper, respectively) was therefore performed in non-splenectomized patients and normal controls in the attempt to verify if Osmoscan coupled parameters differently clustered depending on diseases (Figure 4). Distinguishable clusters were observed for HS, HSt, GPI patients and normal controls. Also patients affected by CDAII and HE clustered, however falling in the normal/HS gates for Omin/EImin, and Ohyper/EIhyper regions and in the HS gate for the EImax/Omax.

### Differential diagnosis between CDAII and HS

To establish the performance of the Osmoscan parameters in discriminating HS between normal controls and patients with other hemolytic anemias ROC curve analysis was performed for Omin, EImax, Omax, and AUC (Table 5).

**Table 5:** Performance of the Osmoscan parameters in discriminating hereditary spherocytosis (HS) between normal control and patients with other membrane defects.

	**HS vs. Normal**	**HS vs. All^§^**	HS vs. Hst^*^	**HS vs. HE**	**HS vs. CDAII**
	**116/170**	**116/257**	**116/6**	**116/15**	**116/16**
Omin	**AUC** = **0.989**	**AUC** = **0.960**	**AUC** = **1.000**	AUC = 0.793	**AUC** = **0.944**
	**(0.980–0.998)**	**(0.942–0.978)**	–	(0.657–0.929)	**(0.988–1.000)**
	**Cut-off** = **153**	**Cut-off** = **157**	**Cut-off** = **140**	Cut-off = 155	**Cut-off** = **157**
	**Se** = **0.95**	**Se** = **0.88**	**Se** = **1.00**	Se = 0.89	**Se** = **0.88**
	**Sp** = **0.94**	**Sp** = **0.93**	**Sp** = **1.00**	Sp = 0.60	**Sp** = **0.94**
EImax	**AUC** = **0.993**	AUC = 0.920	AUC = 0.840	AUC = 0.526	AUC = 0.607
	**(0.983–1.000)**	(0.893–0.947)	(0.671–1.000)	(0.378–0.674)	(0.417–0.797)
	**Cut-off** = **0.56**	Cut-off = 0.54	Cut-off = 0.54	Cut-off = 0.54	Cut-off = 0.51
	**Se** = **0.84**	Se = 0.61	Se = 0.61	Se = 0.59	Se = 0.35
	**Sp** = **0.99**	Sp = 0.89	Sp = 0.67	Sp = 0.27	Sp = 0.56
Ohyper	AUC = 0.706	AUC = 0.718	**AUC** = **0.953**	AUC = 0.731	AUC = 0.617
	(0.638–0.775)	(0.655–0.781)	**(0.899–1.000)**	(0.612–0.850)	(0.528–0.726)
	Cut-off = 475	Cut-off = 475	**Cut-off** = **417**	Cut-off = 475	Cut-off = 461
	Se = 0.80	Se = 0.80	**Se** = **0.85**	Se = 0.80	Se = 0.70
	Sp = 0.24	Sp = 0.33	**Sp** = **1.00**	Sp = 0.40	Sp = 0.25
Area	**AUC** = **0.997**	**AUC** = **0.975**	**AUC** = **0.991**	AUC = 0.740	AUC = 0.893
	**0.994–1.000)**	**(0.962–0.987)**	**(0.975–1.000)**	(0.619–0.861)	(0.817–0.968)
	**Cut-off** = **114**	**Cut-off** = **119**	**Cut-off** = **130**	Cut-off = 112	Cut-off = 119
	**Se** = **0.66**	**Se** = **0.72**	**Se** = **0.97**	Se = 0.56	Se = 0.72
	**Sp** = **0.99**	**Sp** = **0.95**	**Sp** = **0.67**	Sp = 0.67	Sp = 0.75

*AUC, area under the ROC curve; 95% confidence interval (in brackets), cut-off, optimal cut-off calculated by Liu's method; Se, sensitivity and Sp, specificity at the calculated cut-off. For Omin, values higher than cut-off were classified as “positive,” for EImax, Ohyper, and AREA, values lower than cut-off were classified as “positive.” Significant analysis are reported in bold*.

* PIEZO1 mutations;

§ *All non-HS cases (normal subjects included)*.

From the analysis between HS and controls the best parameters were as follows: Omin (AUC 0.989, cut-off = 153), EImax (AUC 0.993, cut-off = 0.56), and AUC (AUC 0.997, cut-off = 114). Similar performances were observed between HS and all non-HS patients: Omin (AUC 0.960, cut-off = 157), and AUC (AUC 0.975, cut-off = 119).

As expected, the best parameters to discriminate HS from HSt were Omin (AUC 1.000, cut-off = 140), Ohyper (AUC 0.953, cut-off = 417), and AUC (AUC 0.991, cut-off = 130). On the contrary, a lower sensitivity and specificity was found in the analysis of HS/HE, thus not allowing establishing diagnostic cut-off. Considering the analysis between HS and CDAII we found that the best parameter to discriminate the two diseases was Omin (AUC 0.944, cut-off = 157).

Moreover, in the attempt to verify whether the combination of ektacytometric and hematologic parameters might improve differential diagnosis between HS and CDAII, we combined Osmoscan and hematologic parameters (data not shown). Omin and absolute reticulocyte number showed the best performances in discriminating the two diseases. We chose the cut-off value of 157 mOsm/kg for Omin as determined through the ROC curve, combined with the commonly used cut-off value of 150 × 10^9^/L for reticulocytes (Russo et al., 2014; King et al., 2015; Bianchi et al., 2016). As reported in Table 6, we observed that when Omin and reticulocyte count were both high, none of patients had CDAII with a reasonably narrow confidence interval. When both indexes were low, all patients had CDAII, but with large uncertainty due the limited number of subjects. A high Omin with low reticulocyte count indicated a low probability (23%) of CDAII. Finally, we had only two patients with high reticulocytes count and low Omin, both with HS.

**Table 6:** Combination of cut-off values of Omin and reticulocytes number for the differential diagnosis of HS and CDAII.

**Reticulocyte number**	**Omin (mOsm/kg)**	
	**≥157**	**<157**
≥150 × 10^9^/L	0/72 = 0%	0/2 = 0%
	95% CI: 0–5%	95% CI: 0–84%
<150 × 10^9^/L	7/30 = 23%	8/8 = 100%
	95% CI: 10–42%	95% CI: 63–100%

*Automated reticulocyte number was available for 97 HS and 15 CDAII cases. CDAII/total number patients for each category is reported and expressed as %*.

## Discussion

This is a monocentric study aimed to evaluate the diagnostic efficiency of the new generation LoRRca MaxSis ektacytometer in an extensive series of congenital hemolytic anemias of various type, including a substantial number of CDAII and enzyme deficiencies never or rarely investigated so far by this technique. The ektacytometric analysis was included in the diagnostic workflow, allowing correlating Osmoscan parameters with the results of all the routine laboratory investigations usually adopted for the diagnosis of hemolytic anemias (Bianchi et al., 2012; King et al., 2015).

Among the methods proposed for the diagnosis of hemolytic anemias, ektacytometry is certainly one of the most interesting due to its versatility, being able to discriminate different defects by a single analysis (Da Costa et al., 2013; King et al., 2015), and his repeatability and reproducibility, enabling an easy standardization in specialized laboratories, as recently reported by other groups (Da Costa et al., 2016; Lazarova et al., 2017; Llaudet-Planas et al., 2018).

Ektacytometry can detect with high sensitivity multiple changes in cellular properties, obtaining information that by conventional methods would require several different types of measurements. In particular, by studying normal cells in which water content and membrane surface area had been selectively modified, it was observed that the resulting curve was a balance in the adjustment of surface area-to-volume ratio (S/V) and intracellular viscosity: an inverse correlation was obtained between MCHC and Ohyper-values together with a tight correlation (correlation coefficient of 0.985) between Omin-values and the osmolality at which hemolysis reached 50% in an osmotic fragility assay (Clark et al., 1983). We therefore investigated correlation of single Osmoscan parameters with red cells indexes and the results of osmotic fragility tests and EMA binding. Omin-values obtained by all the patients, independently from the disease, showed a significant correlation with all the osmotic fragility tests and EMA-binding test. Moreover, MCHC was correlated with Ohyper-values. These data are particularly interesting because obtained from heterogeneous cell populations instead from *in vitro* artificially manipulated red cells (Clark et al., 1983). Despite the correlations observed, these data don't permit to establish diagnostic cut-offs for chronic hemolytic anemias.

To evaluate the diagnostic power of LoRRca MaxSis for hereditary hemolytic anemias, we established in each disease the range values of EImax, Omin, Ohyper, AUC, and their derived parameters (EImin, Omax, EIhyper). In most of the groups of diseases we detected statistically significant differences with normal controls, suggesting that the analysis of a single parameter could help in addressing the diagnosis, as already reported in previous series (Lazarova et al., 2017). This is particularly true for DHSt caused by mutation in *PIEZO1* gene where the typical shape of the curve gives precise diagnostic information, although a newly identified subgroup of stomatocytosis caused by R352H mutation in *KCNN4* gene displays a normal Osmoscan curve (Fermo et al., 2017). In line with previous studies (Llaudet-Planas et al., 2018), also ROC curve analysis led us to identify the osmoscan parameters with the best performance in discriminating HS from normal controls and other kind of hemolytic anemias. However, differences in optimal cut-off, sensitivity, and specificity for single parameters were observed. This could be partially explained by differences in the analyzed cohorts of patients. Inter-laboratory standardization would be useful to establish diagnostic cut-off.

On the contrary, overlaps in values were noted among other hemolytic anemias making not possible differential diagnosis by the analysis of Osmoscan parameters alone. In particular, in our series overlaps were present among data obtained from HS, CDAII, and HE patients.

As regards CDAII, it is worth mentioning that this disorder mimics HS both in terms of clinical presentation and laboratory features, and often requires SDS-PAGE analysis or molecular characterization to be identified (Bianchi et al., 2012; King et al., 2015). We analyzed for the first time a large group of CDAII patients by LoRRca MaxSis: the Osmoscan curve *per se* doesn't allow differential diagnosis, being normal in non-splenectomised CDAII patients and overlapping with HS in the splenectomised ones. We therefore performed the ROC curve of all the Osmoscan and hematologic data looking for the best combination of parameters capable of differentiating CDAII. The cut-off values of 150 × 10^9^/L absolute reticulocyte number and 157 mOsm/kg Omin showed the best performance. Interestingly by this approach we were able to discriminate HS from CDAII with a reasonably narrow confidence interval. The analysis of these parameters on larger series of patients will enable to verify the usefulness of this approach.

Osmoscan analysis of a very large group of different enzymopathies associated with chronic hemolytic anemia led us to identify an unexpected characteristic curve in all the GPI deficient patients tested, offering a first step laboratory screening for this rare enzyme defect. The reason why in GPI deficient patients Ohyper is drastically shifted to the right is unknown and can be only partially attributed to the increased MCV-values observed in these subjects (median MCV 112 fL, range 81–127).

Since Osmoscan curve results by the analysis of the entire red cell population, it is important to underline that concomitance of different defects, as the co-presence of beta-thalassemia trait and splenectomy, may alter the curve shape representing a possible cause of misdiagnosis.

In conclusion, the use of LoRRca MaxSis osmoscan, providing simultaneous information on the major RBC properties (cell geometry, viscosity, and deformability; Da Costa et al., 2016), is therefore particularly indicated in specialized laboratories to be performed as an intermediate step between the first screening (including clinical information, abnormal marker of hemolysis, and red cell morphology) and more specific diagnostic tests. Diagnostic cut-offs have been established, particularly for HS, in line with Llaudet-Planas et al. (2018). However, it is important to underline that in routine practice Osmoscan analysis alone is not sufficient to reach a diagnosis that have to be confirmed by specific second level tests.

## Author contributions

AnZ performed the analysis, analyzed the results, and prepared the manuscript; CV and AM performed hematologic testings; DC performed statistical analysis; EF and PB performed molecular testing, analyzed the results, and prepared the manuscript; WB and AlZ clinical information of patients follow-up and critical revision of the manuscript; AC critical revision of the manuscript.

### Conflict of interest statement

The authors declare that the research was conducted in the absence of any commercial or financial relationships that could be construed as a potential conflict of interest.

## Supplementary material

Supplementary Figure 1 Correlation between Osmoscan parameters and laboratory and hematologic data. GLT, standard glycerol lysis test; AGLT, acidified glycerol lysis test; EMA, eosin-5-maleimide.

## References

[B1] Badens C. Guizouarn H. (2016). Advances in understanding the pathogenesis of the red cell volume disorders. Br. J. Haematol. 174, 674–685. 10.1111/bjh.14197 27353637

[B2] Baskurt O. K. Hardeman M. R. Uyuklu M. Ulker P. Cengiz M. Nemeth N. (2009). Parameterization of red blood cell elongation index – shear stress curves obtained by ektacytometry, Scand. J. Clin. Lab. Invest. 69, 777–788. 10.3109/00365510903266069 19929721

[B3] Baskurt O. K. Meiselman H. J. (2004). Analyzing shear stress–elongation index curves: comparison of two approaches to simplify data presentation. Clin. Hemorheol. Microcirc. 31, 23–30. 15272150

[B4] Beutler E. (1984). Red Cell Metabolism: A Manual of Biochemical Methods. New York, NY: Grune & Stratton, Inc.

[B5] Bianchi P. Fermo E. Vercellati C. Marcello A. P. Porretti L. Cortelezzi A.. (2012). Diagnostic power of laboratory tests for hereditary spherocytosis: a comparison study in 150 patients grouped according to molecular and clinical characteristics. Haematologica 97, 516–523. 10.3324/haematol.2011.052845 22058213 PMC3347664

[B6] Bianchi P. Schwarz K. Högel J. Fermo E. Vercellati C. Grosse R.. (2016). Analysis of a cohort of 101 CDAII patients: description of 24 new molecular variants and genotype-phenotype correlations. Br. J. Haematol. 175, 696–704. 10.1111/bjh.14271 27471141

[B7] Clark M. R. Mohandas N. Shohet S. B. (1983). Osmotic gradient ektacytometry: comprehensive characterization of red cell volume and surface maintenance. Blood 61, 899–910. 6831052

[B8] Dacie J. V. Lewis S. M. (2001). Practical Haematology, 9th Edn London: Churchill Livingston.

[B9] Da Costa L. Galimand J. Fenneteau O. Mohandas N. (2013). Hereditary spherocytosis, elliptocytosis, and other red cell membrane disorders. Blood Rev. 27, 167–178. 10.1016/j.blre.2013.04.003 23664421

[B10] Da Costa L. Suner L. Galimand J. Bonnel A. Pascreau T. Couque N.. (2016). Diagnostic tool for red blood cell membrane disorders: assessment of a new generation ektacytometer. Blood Cells Mol. Dis. 56, 9–22. 10.1016/j.bcmd.2015.09.001 26603718 PMC4811191

[B11] Fairbanks G. Steck T. L. Wallach D. F. H. (1971). Electrophoretic analysis of bthe major polypeptides of the human erythrocyte membrane. Biochemistry 10, 2606–2617. 10.1021/bi00789a030 4326772

[B12] Fermo E. Bogdanova A. Petkova-Kirova P. Zaninoni A. Marcello A. P. Makhro A.. (2017). ‘Gardos Channelopathy': a variant of hereditary Stomatocytosis with complex molecular regulation. Sci. Rep. 7:1744. 10.1038/s41598-017-01591-w 28496185 PMC5431847

[B13] Glogowska E. Gallagher P. G. (2015). Disorders of erythrocyte volume homeostasis. Int. J. Lab. Hematol. 37, 85–91. 10.1111/ijlh.12357 25976965 PMC4435826

[B14] He Y. Jia S. Dewan R. K. Liao N. (2017). Novel mutations in patients with hereditary red blood cell membrane disorders using next-generation sequencing. Gene 627, 556–562. 10.1016/j.gene.2017.07.009 28694211

[B15] King M. J. Behrens J. Rogers C. Flynn C. Greenwood D. Chambers K. (2000). Rapid flow cytometric test for the diagnosis of membrane cytoskeleton-associated haemolytic anaemia. Br. J. Haematol. 111, 924–933. 10.1111/j.1365-2141.2000.02416.x 11122157

[B16] King M. J. Garçon L. Hoyer J. D. Iolascon A. Picard V. Stewart G.. (2015). ICSH guidelines for the laboratory diagnosis of nonimmune hereditary red cell membrane disorders. Int. J. Lab. Hematol. 37, 304–325. 10.1111/ijlh.12335 25790109

[B17] King M. J. Zanella A. (2013). Hereditary red cell membrane disorders and laboratory diagnostic testing. Int. J. Lab. Hematol. 35, 237–243. 10.1111/ijlh.12070 23480868

[B18] Koralkova P. van Solinge W. W. van Wijk R. (2014). Rare hereditary red blood cell enzymopathies associated with hemolytic anemia - pathophysiology, clinical aspects, and laboratory diagnosis. Int. J. Lab. Hematol. 36, 388–397. 10.1111/ijlh.12223 24750686

[B19] Laemmli U. K. (1970). Cleavage of structural proteins during the assembly of the head of bacteriophage T4. Nature 227, 680–685. 10.1038/227680a0 5432063

[B20] Lazarova E. Gulbis B. Oirschot B. V. van Wijk R. (2017). Next-generation osmotic gradient ektacytometry for the diagnosis of hereditary spherocytosis: interlaboratory method validation and experience. Clin. Chem. Lab. Med. 55, 394–402. 10.1515/cclm-2016-0290 27559691

[B21] Liu X. (2012). Classification accuracy and cut point selection. Stat. Med. 31, 2676–2686. 10.1002/sim.4509 22307964

[B22] Llaudet-Planas E. Vives-Corrons J. L. Rizzuto V. Gómez-Ramírez P. Sevilla Navarro J. Coll Sibina M. T.. (2018). Osmotic gradient ektacytometry: a valuable screening test for hereditary spherocytosis and other red blood cell membrane disorders. Int. J. Lab. Hematol. 40, 94–102. 10.1111/ijlh.12746 29024480

[B23] Mariani M. Barcellini W. Vercellati C. Marcello A. P. Fermo E. Pedotti P.. (2008). Clinical and hematologic features of 300 patients affected by hereditary spherocytosis grouped according to the type of the membrane protein defect. Haematologica 93, 1310–1317. 10.3324/haematol.12546 18641031

[B24] Mohandas N. Gallagher P. G. (2008). Red cell membrane: past, present, and future. Blood 112, 3939–3948. 10.1182/blood-2008-07-161166 18988878 PMC2582001

[B25] Perrotta S. Gallagher P. G. Mohandas N. (2008). Hereditary spherocytosis. Lancet 372, 1411–1426. 10.1016/S0140-6736(08)61588-3 18940465

[B26] Russo R. Gambale A. Langella C. Andolfo I. Unal S. Iolascon A. (2014). Retrospective cohort study of 205 cases with congenital dyserythropoietic anemia type II: definition of clinical and molecular spectrum and identification of new diagnostic scores. Am. J. Hematol. 89, E169–E175. 10.1002/ajh.23800 25044164

[B27] StataCorp (2017). Stata Statistical Software: Release 15. College Station, TX: StataCorp LP.

