# Supplementary figures and images for: Use of Laser Assisted Optical Rotational Cell Analyzer (LoRRca MaxSis) in the Diagnosis of RBC Membrane Disorders, Enzyme Defects, and Congenital Dyserythropoietic Anemias: A Monocentric Study on 202 Patients
Source: Front Physiol. 2018 Apr 27;9:451. doi: 10.3389/fphys.2018.00451 (PMC5934481; doi:10.3389/fphys.2018.00451)

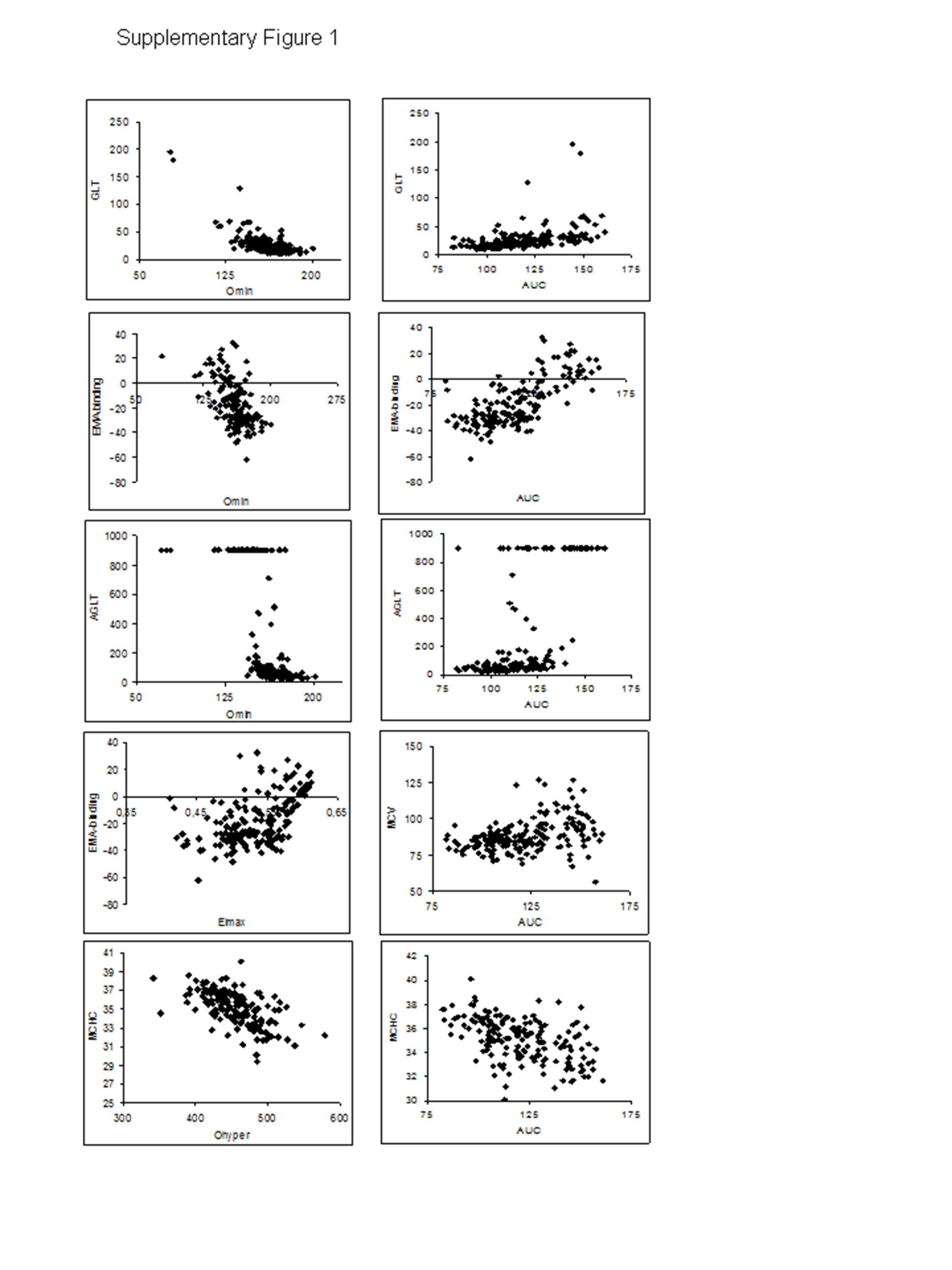

Supplement: Supplementary Figure 1 — Correlation between Osmoscan parameters and laboratory and hematologic data. GLT, standard glycerol lysis test; AGLT, acidified glycerol lysis test; EMA, eosin-5-maleimide. [file Image_1.TIF]
